# Peritoneal Dialysis-Associated Peritonitis Rates in the Outpatient and Hospital Setting Among Incident Dialysis Patients With Medicare, 2009–2018

**DOI:** 10.1016/j.xkme.2024.100931

**Published:** 2024-11-15

**Authors:** Christopher Knapp, Shuling Li, Chuanyu Kou, James B. Wetmore, Kirsten L. Johansen

**Affiliations:** 1 Chronic Disease Research Group, Minneapolis, MN; 2 Hennepin Healthcare System, Department of Medicine, Division of Nephrology, Minneapolis, MN; 3 Department of Medicine, University of Minnesota Medical School, Minneapolis, MN

To the Editor:

Peritonitis is a leading cause of morbidity for peritoneal dialysis (PD) patients, with 10% to 20% of events leading to PD cessation.^1^^,^^2^ According to the United States Renal Data System (USRDS), rates of hospitalization for peritonitis have decreased by more than 50% in the US since 2009.^3^ However, other US studies reported a higher rate of peritonitis hospitalization than is reported by USRDS, raising the possibility that USRDS data on peritonitis as the primary cause of hospitalization may not accurately reflect the true burden of peritonitis.^4^ Moreover, it is unclear if this trend toward improved peritonitis hospitalization rates has been matched by improvements in outpatient peritonitis rates or has occurred partially as a result of shifting peritonitis treatment to the outpatient setting. We therefore used a broader definition of peritonitis hospitalization and used antibiotics claims data to define outpatient peritonitis events to more fully examine how peritonitis rates changed for people initiating PD in the United States during the 2009–2018 period.

We used data from the USRDS to identify a cohort of all adults starting PD with Medicare fee-for-service insurance on their end-stage renal disease first service date from 2009 to 2018. Patients were followed for up to 2 years after dialysis initiation or until they experienced death, transplant, recovery of kidney function, conversion to hemodialysis, or discontinuation of fee-for-service coverage. We excluded patients who initiated dialysis in 2019 to 2020 from our analysis because of the effects of the coronavirus disease (COVID)-19 pandemic on PD outcomes.^5^ Peritonitis episodes were grouped according to the setting of the peritonitis event (hospitalization vs outpatient). Item S1 in the supplementary material discusses the research ethics committee and consent process.

Hospitalization with peritonitis was defined as a hospitalization with a claim for peritonitis in any diagnosis position. An outpatient peritonitis event was identified through dialysis facility claims for antibiotics commonly used to treat peritonitis. To distinguish peritonitis events from contamination events, qualifying claims had to be on 2 or more days within a week, have at least 2 antibiotics on 1 day, or have an associated peritonitis-related code. We defined a peritonitis hospitalization or outpatient event as independent if the event started more than 4 weeks after the beginning date of a prior event and 7 days after the end date of the prior event, as defined by admission and discharge dates for hospital events and dates of the first and last dose of antibiotic for outpatient events. Recurrent hospitalizations or antibiotic prescriptions within these parameters were defined as a single independent event.

We reported unadjusted rates as the number of peritonitis events per 100 patient-years of time at risk for yearly cohorts. We then used Poisson regression models to estimate rate ratios of peritonitis events over time. We modeled calendar year as a continuous variable to estimate the relative risks (RRs) for these events per 2 years over the study period. Unadjusted models included only calendar year as a covariate. Adjusted models additionally included patent demographic characteristics, geographic factors, facility PD census, and medical comorbid conditions as covariates (listed in Table S1). We reported results with 95% confidence intervals (CIs) and tested for statistical significance at a two-tailed α level of 0.05.

Our study population included 40,059 patients. The average age was 64 years. In total, 19% were Black, 65% were White, and 77% lived in urban areas (Table S1). The size of the yearly incident PD cohorts in our population grew by 160% over the study period. Patient’s median follow up time was 523 days. The unadjusted rate of combined peritonitis events for the study population fell from 58.1 per 100 patient-years in 2009 to 30.7 in 2018 (Fig 1). The adjusted rate ratio for hospitalization with peritonitis decreased by 11% per 2-year period (RR per two years 0.89, 95% CI 0.87-0.90), nearly identical to the decrease observed in the rate ratio for outpatient peritonitis (RR 0.88, 95% CI 0.87-0.89) and the ratio for the combined outcome (RR 0.88, 95% CI 0.87-0.89) (Table 1).

**Figure 1:**
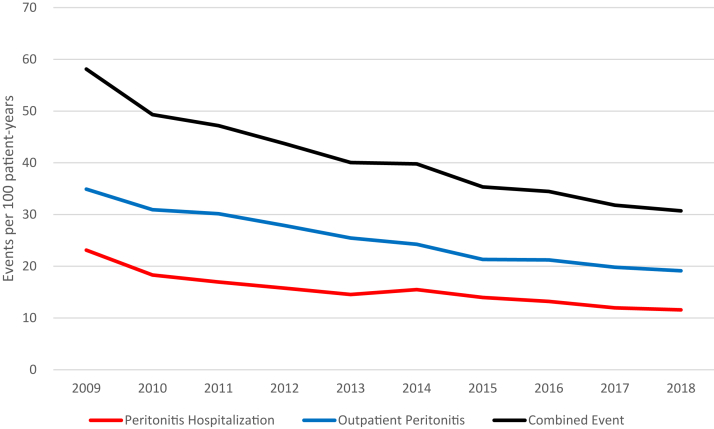
**Unadjusted rate of peritonitis among incident ESRD patients undergoing peritoneal dialysis, by setting of peritonitis event, 2009–2018**. Rates are expressed as number of events per 100 patient years. Events in the inpatient setting, outpatient setting, and combined events are shown.

**Table 1:** Unadjusted and Adjusted Rate Ratios of Peritonitis Hospitalization, Outpatient Peritonitis, and Combined Event Among Patients With Kidney Failure Initiating Peritoneal Dialysis, per Two Years of the Study Period.

Outcome	Unadjusted RR, per 2 Years^a^ (95% CI)	*P* Value for Trend	Adjusted RR, per 2 years^1^	*P* Value for Trend
**Peritonitis Hospitalization**	0.877 (0.863,0.891)	<.001	0.888 (0.873, 0.902)	<.001
**Outpatient Peritonitis**	0.873 (0.862, 0.884)	<.001	0.878 (0.867, 0.889)	<.001
**Combined Event**	0.874 (0.866,0.883)	<.001	0.882 (0.873, 0.891)	<.001

a Both models included calendar year. The adjusted model also included age group, sex, race/ethnicity, social deprivation index and rural/urban status of the patient’s ZIP code, medical comorbid conditions, physical deconditioning, BMI, census region, and PD facility size as additional covariates.

We found that peritonitis rates improved universally, regardless of inpatient or outpatient setting. This improvement is notable in light of the rapid increase in the PD population during the study period, especially given that technique survival (ie, avoidance of conversion from PD to in-center hemodialysis), another key indicator for PD patients linked with peritonitis, did not improve nearly as much over the same period.^2^^,^^3^^,^^6^ It is possible that more widespread use of prophylactic topical antibiotics or increased use of automated PD instead of continuous ambulatory PD in this period were partially responsible for this improvement.^7^, ^8^, ^9^, ^10^ However, PD technology and techniques were otherwise largely unchanged over this time, so the divergence of peritonitis and technique survival outcomes is surprising and merits further study. Our study was limited to the Medicare fee-for-service population, which is older and less diverse than the PD population as a whole.^3^ Even so, our study offers evidence that the burden of peritonitis has significantly decreased for individuals who perform PD.

## Supplementary Materials

Supplementary File (PDF) Item S1; Table S1.
