# Supplementary material for: Peritoneal Dialysis-Associated Peritonitis Rates in the Outpatient and Hospital Setting Among Incident Dialysis Patients With Medicare, 2009–2018
Source: Kidney Med. 2024 Nov 15;7(1):100931. doi: 10.1016/j.xkme.2024.100931 (PMC11697115; doi:10.1016/j.xkme.2024.100931)
Supplement: Supplementary File (PDF) — Item S1; Table S1. [file mmc1.pdf]

Item S1: Detailed Methods

This research was deemed to not involve human subjects and was thus exempted from need for IRB approval by the IRB of Hennepin Healthcare systems in Minneapolis, MN. Because no human subjects were involved, the requirement for informed consent was waived.

Table S1. Demographic Characteristics, Incident PD Patients With Medicare Fee-for-Service Insurance at Dialysis Initiation, 2009-2018

Characteristic	2009-2018 combined	2009-2011	2012-2014	2015-2017	2018
**Total, n**	40,059	9,594	12,586	13,071	4,808
**Age group**					
18-44 years, n (%)	5,190 (13.0)	1,379 (14.4)	1,730 (13.7)	1,519 (11.6)	562 (11.7)
45-64 years, n (%)	11,963 (29.9)	3,092 (32.2)	4,000 (31.8)	3,632 (27.8)	1,239 (25.8)
65-74 years, n (%)	13,129 (32.8)	2,851 (29.7)	3,880 (30.8)	4,676 (35.8)	1,722 (35.8)
75+ years, n (%)	9,777 (24.4)	2,272 (23.7)	2,976 (23.6)	3,244 (24.8)	1,285 (26.7)
**Race/Ethnicity**					
Black, n (%)	7,577 (18.9)	1,878 (19.6)	2,516 (20.0)	2,374 (18.2)	809 (16.8)
Hispanic, n (%)	3,974 (9.9)	935 (9.7)	1,330 (10.6)	1,224 (9.4)	485 (10.1)
Other, n (%)	2,379 (5.9)	534 (5.6)	726 (5.8)	822 (6.3)	297 (6.2)
White, n (%)	26,129 (65.2)	6,247 (65.1)	8,014 (63.7)	8,651 (66.1)	3,217 (66.9)
**Rural/Urban status**[b]					
Urban, n (%)	30,939 (77.2)	7,340 (76.5)	9,801 (77.9)	10,147 (77.6)	3,651 (75.9)
**Social Deprivation Index group**[c]					
1-33, n (%)	11,595 (28.9)	2,702 (28.1)	3,504 (27.8)	3,920 (30.0)	1,469 (30.6)
34-66, n (%)	14,273 (35.6)	3,413 (35.6)	4,504 (35.8)	4,633 (35.4)	1,723 (35.8)
67-100, n (%)	14,191 (35.4)	3,479 (36.2)	4,578 (36.4)	4,518 (34.6)	1,616 (33.6)
**Female, n (%)**	17,306 (43.2)	4,247 (44.3)	5,467 (43.4)	5,496 (42.0)	2,096 (43.6)
**Comorbid conditions**					
ASHD, n (%)	5,522 (13.8)	1,743 (18.1)	1,755 (13.9)	1,517 (11.6)	507 (10.5)

CHF, n (%)	7,688 (19.2)	2,111 (22.0)	2,503 (19.9)	2,225 (17.0)	849 (17.7)
CVA/TIA, n (%)	2,772 (6.9)	759 (7.9)	844 (6.7)	864 (6.6)	305 (6.3)
PVD, n (%)	3,457 (8.6)	1,122 (11.7)	1,051 (8.4)	948 (7.3)	336 (7.0)
Other cardiac, n (%)	6,003 (15.0)	1,391 (14.4)	1,856 (14.7)	1,998 (15.3)	758 (15.8)
Cancer, n (%)	2,616 (6.5)	640 (6.7)	752 (6.0)	878 (6.7)	346 (7.2)
COPD, n (%)	2,303 (5.7)	592 (6.1)	755 (6.0)	709 (5.4)	247 (5.1)
Diabetes, n (%)	21,192 (52.9)	4,851 (50.6)	6,635 (52.7)	7,054 (54.0)	2,652 (55.2)
**Physical Deconditioning,[d] n (%)**	2,553 (6.4)	643 (6.7)	792 (6.3)	816 (6.2)	302 (6.3)
**BMI**					
<18.5, n (%)	864 (2.2)	205 (2.1)	266 (2.1)	294 (2.2)	99 (2.1)
18.5-24.9	10,687 (26.7)	2,710 (28.2)	3,325 (26.4)	3,378 (25.8)	1,274 (26.5)
25.0-29.9	12,761 (31.9)	3,047 (31.8)	4,073 (32.4)	4,178 (32.0)	1,463 (30.4)
>=30	15,747 (39.3)	3,632 (37.9)	4,922 (39.1)	5,221 (39.9)	1,972 (41.0)
**Region**					
Northeast, n (%)	4,309 (10.8)	1,053 (11.0)	1,336 (10.6)	1,385 (10.6)	535 (11.1)
Midwest, n (%)	8,995 (22.5)	2,129 (22.2)	2,788 (22.2)	3,013 (23.0)	1,065 (22.2)
South, n (%)	19,011 (47.5)	4,625 (48.2)	6,030 (47.9)	6,112 (46.8)	2,244 (46.7)
West, n (%)	7,744 (19.3)	1,787 (18.6)	2,432 (19.3)	2,561 (19.6)	964 (20.0)
**Facility size**					
<26 PD patients, n (%)	20,365 (50.8)	4,969 (51.8)	6,385 (50.7)	6,645 (50.8)	2,366 (49.2)
>=26 PD patients, n (%)	19,694 (49.2)	4,625 (48.2)	6,201 (49.3)	6,426 (49.2)	2,442 (50.8)

[a] Based on Medical Evidence Form.

[b]Defined by Rural Urban Commuting Area code (Economic Research Service, 2020) of 3 or less, as determined by patient ZIP code

[c] Social Deprivation Index Score (Butler, 2013) is determined by patient ZIP code. 1= least deprived. 100= most deprived

[d] Defined as answering yes to any of three questions on Medical Evidence Form regarding inability to transfer, inability to ambulate, or needing assistance with activities of daily living.
